# Bioinformatics tools for marine biotechnology: a practical tutorial with a metagenomic approach

**DOI:** 10.1186/s12859-020-03560-z

**Published:** 2020-08-21

**Authors:** Ludovica Liguori, Maria Monticelli, Mariateresa Allocca, Maria Vittoria Cubellis, Bruno Hay Mele

**Affiliations:** 1 Dipartimento di Scienze e Tecnologie Ambientali, Biologiche e Farmaceutiche, Università degli Studi della Campania “Luigi Vanvitelli”, 81100 Caserta, Italy; 2 Istituto di Chimica Biomolecolare –CNR, 80078 Pozzuoli, Italy; 3 Dipartimento di Biologia, Università Federico II, 80126 Naples, Italy; 4 Biology and Evolution of Marine Organisms Department, Stazione Zoologica Anton Dohrn, Villa Comunale, 80121 Naples, Italy; 5 Integrative Marine Ecology Department, Stazione Zoologica Anton Dohrn, Villa Comunale, 80121 Naples, Italy

**Keywords:** Marine biotechnology, Graduate education, Computer-based learning

## Abstract

**Background:**

Bioinformatics has pervaded all fields of biology and has become an indispensable tool for almost all research projects. Although teaching bioinformatics has been incorporated in all traditional life science curricula, practical hands-on experiences in tight combination with wet-lab experiments are needed to motivate students.

**Results:**

We present a tutorial that starts from a practical problem: finding novel enzymes from marine environments. First, we introduce the idea of metagenomics, a recent approach that extends biotechnology to non-culturable microbes. We presuppose that a probe for the screening of metagenomic cosmid library is needed. The students start from the chemical structure of the substrate that should be acted on by the novel enzyme and end with the sequence of the probe. To attain their goal, they discover databases such as BRENDA and programs such as BLAST and Clustal Omega.

Students’ answers to a satisfaction questionnaire show that a multistep tutorial integrated into a research wet-lab project is preferable to conventional lectures illustrating bioinformatics tools.

**Conclusion:**

Experimental biologists can better operate basic bioinformatics if a problem-solving approach is chosen.

## Background

At present any biologists should capitalize on the resources, data and programs, that are available online to make their experimental plans more efficient and cost-effective [1, 2]. For this reason, it is desirable to train students using a problem-solving approach that integrates in silico work into a multidisciplinary experimental project(a few examples [3–13]. We provide a tutorial that was administered to students of the courses of marine and environmental biology.

Biodegradation of environmental pollutants by marine prokaryotic enzymes provides the frame into which the bioinformatics tutorial is inserted. In particular, the experimental project proposed to the students, aims at finding an enzyme that is active on a scaffold commonly found among pollutants and synthetic compounds, a so-called “privileged scaffold” [14]. In this exercise, the scaffold taken into consideration is indole, an N-heterocyclic aromatic pollutant released in the aquatic environment through the industrial wastewater [15].

The students will look for an enzyme with broad specificity that is able to degrade indole and more in general aromatic compounds [16]. As it is widely known, only a small fraction of environmental microbes grow under conventional laboratory conditions [17–19]. For this reason, several authors suggested that metagenomes might be a big reservoir of novel enzymes for applications in biocatalysis, biofuels, and bioremediation (for reviews on this subject [20–23]).

The project consists of two parts: a laboratory part, that we won’t discuss in detail here, to construct a DNA clones library from the metagenomic marine sample, and a bioinformatics part that is the object of this paper.

We presuppose that DNA has been extracted from prokaryotes present in a seawater sample and that a metagenomic library has been constructed in cosmids. In principle, the screening could be carried out testing the enzymatic activity of interest directly. However, assays on plates can be cumbersome and we propose an alternative strategy that takes advantage of the simplicity of DNA colony hybridization. Only after the clone has been identified by a DNA labelled probe and isolated, the activity will be confirmed in the recombinant *E.coli* extracts.

The tutorial focuses on the design of a suitable probe for the screening. It combines information deriving from a highly annotated enzyme database such as BRENDA [24] with data on uncharacterized open reading frames (ORF) deriving from large scale metagenomic sequencing projects.

BRENDA is freely available for academic users and educational purposes [25]. Enzymes are classified according to the catalyzed reactions and well-characterized proteins from different organisms can be found in each class. BRENDA [24] can be searched with a structure-based query as well as with a text-based query. It provides a substructure search algorithm that is very useful to use a scaffold as a query. This choice is very convenient because chemical structures identify molecules uniquely whereas names are not unique. For instance, indole is also known as 1-Benzazole,1H-Benzo [b]pyrrole,1H-Indol,1H-Indole, nomenclature can be even more confusing for other molecules. BRENDA can be searched for several specific information at the same time with “Advanced options”. The tutorial shows how a bacterial enzyme that is active on a scaffold commonly found among pollutants and synthetic compounds can be found. We imagine that the sequence cannot be used as such for colony hybridization of the cosmid library under stringent conditions. Hence we propose to use the enzyme found in BRENDA [24] as a query sequence to look for homologous proteins among uncharacterized ORF from marine metagenomes. This is possible because BLASTp [26] consents to search protein sequences from large environmental sequencing projects such as the Malaspina expedition [27], Global Ocean Sampling (GOS) campaign [28] and Tara Oceans expedition [29].

The tutorial was administered to 23 students with no previous knowledge of bioinformatics who filled a satisfaction test at the end of the exercise.

## Methods

### Aims

This tutorial is intended for laboratory biologists with no previous knowledge of bioinformatics. We envisaged a multistep bioinformatics protocol that is integrated into a project of marine biotechnology. The protocol focuses on the use of four bioinformatics web applications (BLASTp [26], Clustal Omega, Cons, Reverse Translate [30]) and two biochemical databases (UniProt [31] and BRENDA [24]). The learning goals are summarized in Table 1.

**Table 1:** Learning goals

Software/Database Employed	Educational Goal
BRENDA Uniprot BLASTp	Obtain information for a given enzyme class. Obtain the sequence and the annotations for a given protein. Search sequence databases using a protein query.
Clustal Omega	Build phylogenetic trees and multiple alignments.
Cons	Derive a Consensus sequence from a multiple alignment.
Reverse Translate	Derive a nucleotide sequence from an amino acid sequence.

### Experiment overview

**Fig. 1:**
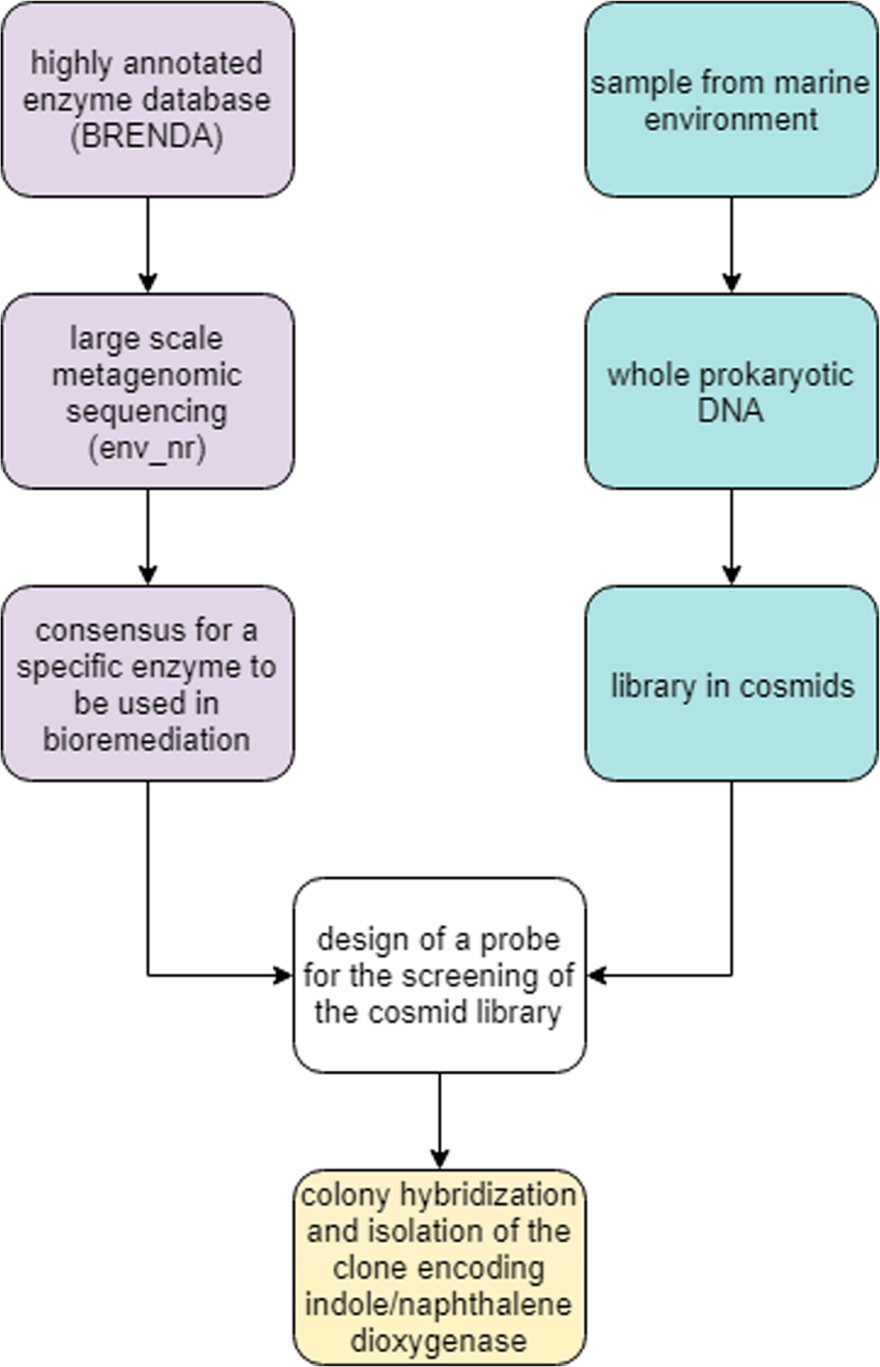
Flowchart of the training. The bioinformatics analysis (purple rectangles) and the wet-lab cosmid library synthesis (light blue rectangles) merge when the probe for the screening is designed (white rectangle) and the colony hybridization is carried out (yellow rectangle)

We presuppose that the students are engaged in a project for the identification of a novel bacterial enzyme from a seawater metagenomic sample. For this purpose, we assume that a library has been constructed in cosmids and must be screened. In this tutorial, we present a bioinformatics protocol to design a probe to isolate the clone of interest (Fig. 1).

In the first step, the students will search BRENDA [24], the enzyme database [25], to find a prokaryotic enzyme that is able to use a given class of pollutants as substrates. BRENDA [24] consents looking among enzyme ligands by chemical similarity and by substructures. In the tutorial, indole is chosen as an example because it represents a privileged scaffold, i.e. its chemically active structure is common to many natural and synthetic compounds with the ability to bind different targets [14]. Unfortunately, it is not (yet) possible to carry out an advanced search drawing a substructure and for this reason, to get an enzyme class that is active on a given substrate, identified by its chemical structure, AND is expressed in bacteria, it is necessary to follow a two-phase, apparently redundant, protocol. In the first phase, the structure is used as the input to get the exact name (i.e. the one used by BRENDA that does not necessarily coincides with the IUPAC name) of the substrate of interest. In the second phase, the exact name and the class of organisms are used with advanced options. Several classes of enzymes are active on molecules that resemble indole. Naphthalene 1,2-dioxygenase has broad specificity and is involved in the degradation of many aromatic compounds [32]. By clicking on the E.C. number the students will get much information that is subdivided into different sections. The most interesting ones for the case under study are those concerning enzyme-ligands interactions where it is possible to learn which are all the possible substrates of this class on enzymes, those concerning organism related information and those related to enzyme structures, where the links to UniProt [https://www.uniprot.org/] [31] are found. The students will not find any Naphthalene 1,2-dioxygenases from a prokaryotic marine organism. At present (March 2019) the only well-characterized prokaryotic enzyme is from *Pseudomonas putida* [33].

In the second step, the sequence of Naphthalene 1,2-dioxygenases from *Pseudomonas putida* will be obtained from UniProt [https://www.uniprot.org/] [31]. *Pseudomonas putida* is evolutionarily distant from the marine prokaryotes that are present in the sample of marine water used to construct the library. The students will look for homologous sequences from marine prokaryotes. They will take advantage of a large number of uncharacterized coding sequences obtained by massive genomic and metagenomic sequence projects. To carry out such an analysis, they will use BLASTp [https://blast.ncbi.nlm.nih.gov/Blast.cgi? PROGRAM = blastp&PAGE_TYPE = BlastSearch&LINK_LOC = blasthome] using Naphthalene 1,2-dioxygenases from *Pseudomonas putida* as the query and limiting the search to the metagenomic proteins deposited in databases (env_nr).

In the third step, a multiple sequence alignment will be carried out using Clustal Omega [https://www.ebi.ac.uk/Tools/msa/clustalo/] [34]. The students will identify conserved regions among the sequences homologous Naphthalene 1,2-dioxygenases from *Pseudomonas putida* and will derive a consensus using Cons [http://www.bioinformatics.nl/cgi-bin/emboss/cons] [35–37].

In the fourth step, the retrotranslation of the aminoacidic consensus sequence will be carried out using [https://www.bioinformatics.org/sms2/rev_trans.html] [30]. The students will learn that retrotranslation does not provide a unique DNA sequence unless the codon most frequently used for each amino acid in prokaryotes is chosen.

The output of this tutorial is a sequence of the probe for the screening by colony hybridization.

### Requirements

All software used is free with a user-friendly interface available. The only requirement is a computer with an Internet connection.

The used software and databases are:

BRENDA, https://www.brenda-enzymes.org/index.php

BLASTP, https://blast.ncbi.nlm.nih.gov/Blast.cgi

UniProt, http://www.uniprot.org/

Clustal Omega, https://www.ebi.ac.uk/Tools/msa/clustalo/

Cons, http://www.bioinformatics.nl/emboss-explorer/

Reverse translate, http://www.bioinformatics.org/sms2/rev_trans.html

### Detailed protocol

#### Step 1. BRENDA: searching the enzyme database to find a microbial enzyme able to degrade indole

Access BRENDA (The Comprehensive Enzyme Information System) at https://www.brenda-enzymes.org/index.php.

**Fig. 2:**
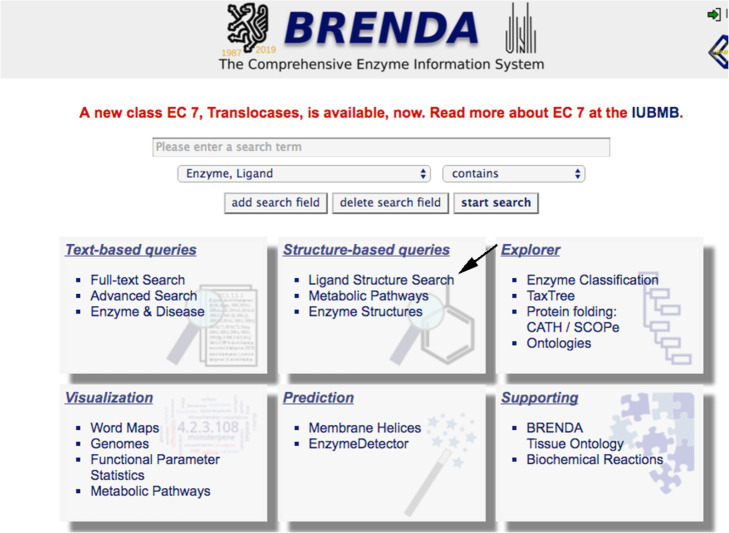
BRENDA’s graphical interface 1. Ligand search can be selected from the Homepage

**Fig. 3:**
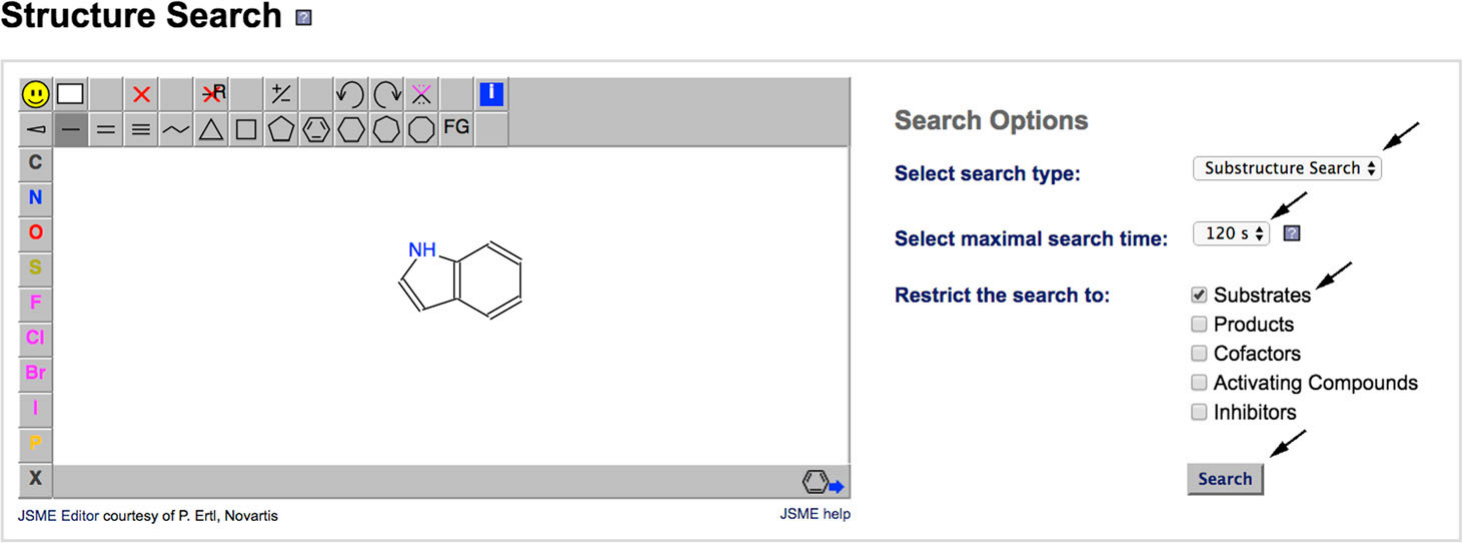
BRENDA’s graphical interface 2. The structure search tool can be chosen to draw chemical scaffolds

Draw the molecule of interest, a bicyclic structure, consisting of a six-membered benzene ring fused to a five-membered pyrrole ring, clicking on “Ligand Structure Search” in the BRENDA homepage (Fig. 2) to obtain the exact name of the compound. A “substructure search” with a maximal search time of 120 s restricted to “Substrates” must be selected before running the search Fig. 3).

**Fig. 4:**
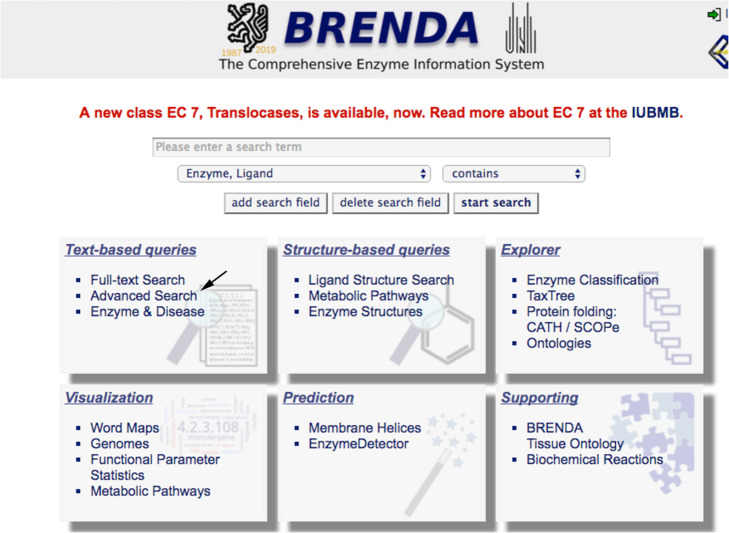
BRENDA’s graphical interface 3. Advanced search can be selected from the Homepage

**Fig. 5:**
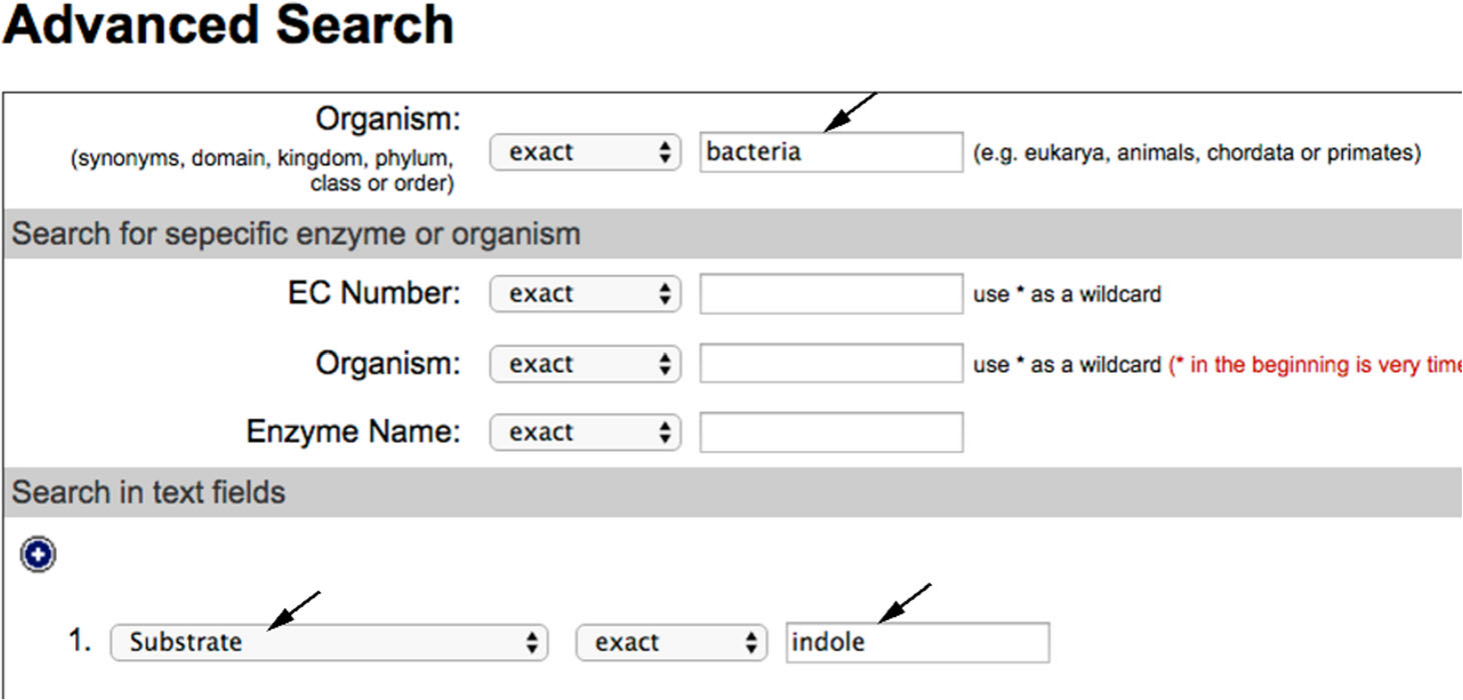
Performing an Advanced Search in BRENDA. The selection of specific terms is shown

You obtain the exact names of several molecules containing the structure you drew, you choose “indole”. Go back to BRENDA homepage and run an advanced search (Fig. 4) filling in the kingdom (bacteria) and type (substrate) boxes and using the exact name of the molecule (indole) (Fig. 5).

**Fig. 6:**
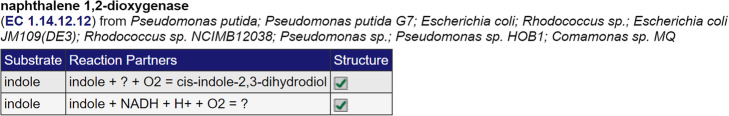
Classes of enzymes acting on indole in BRENDA (inset). Naphthalene 1,2-dioxygenase can be selected among the enzyme classes acting on indole

BRENDA shows a list of all the enzymes in the database that use indole as a substrate. The enzyme Naphthalene 1,2-dioxygenase from *Pseudomonas* has been chosen from the results for this training. It is present in different bacterial species and has a large substrate specificity. Please click on the EC number (Fig. 6) to obtain all the biochemical data about the enzyme.

**Fig. 7:**
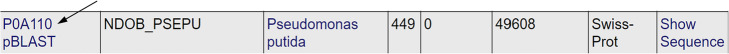
Specific bacterial enzyme in BRENDA. The link to UniProt can be selected for Naphthalene 1,2-dioxygenases from specific proteins

In the section “enzyme structures”, look for “AA sequences” where the links to UniProt [https://www.uniprot.org/] [31] are found (Fig. 7). *Pseudomonas putida* has been chosen as an organism; click on the UniProt code to open the link to the proteins sequences database.

**Fig. 8:**
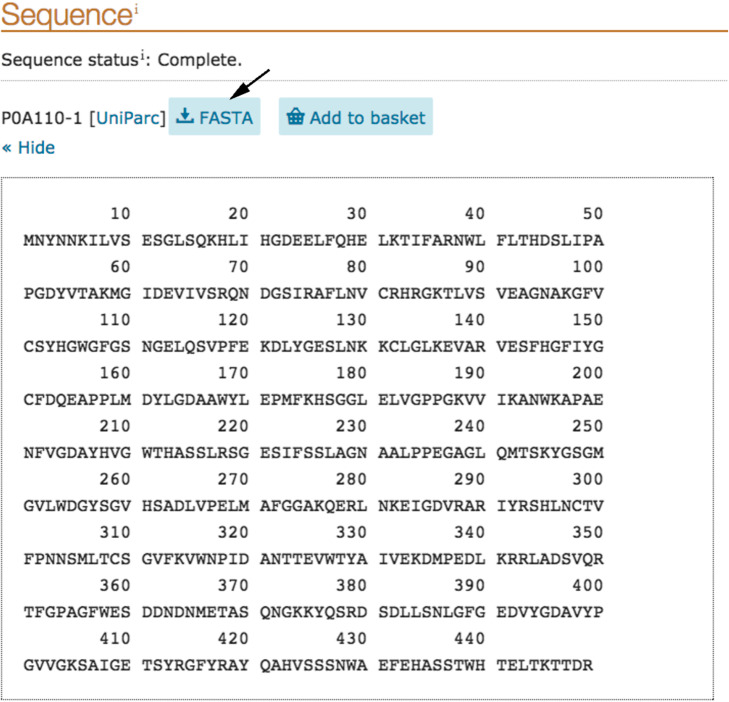
The amino acid sequence of Naphthalene 1,2-dioxygenase in the UniProt database

On the UniProt sheet choose Sequence and download the amino acid sequence in FASTA format (Fig. 8).

#### Step 2. Protein BLAST (BLASTp) to carry out a local alignment to compare an amino acid sequence to the metagenomic proteins deposited into a database

Open a new Internet page with BLAST at https://blast.ncbi.nlm.nih.gov/Blast.cgi and click on “Protein BLAST” to align amino acid sequences.

**Fig. 9:**
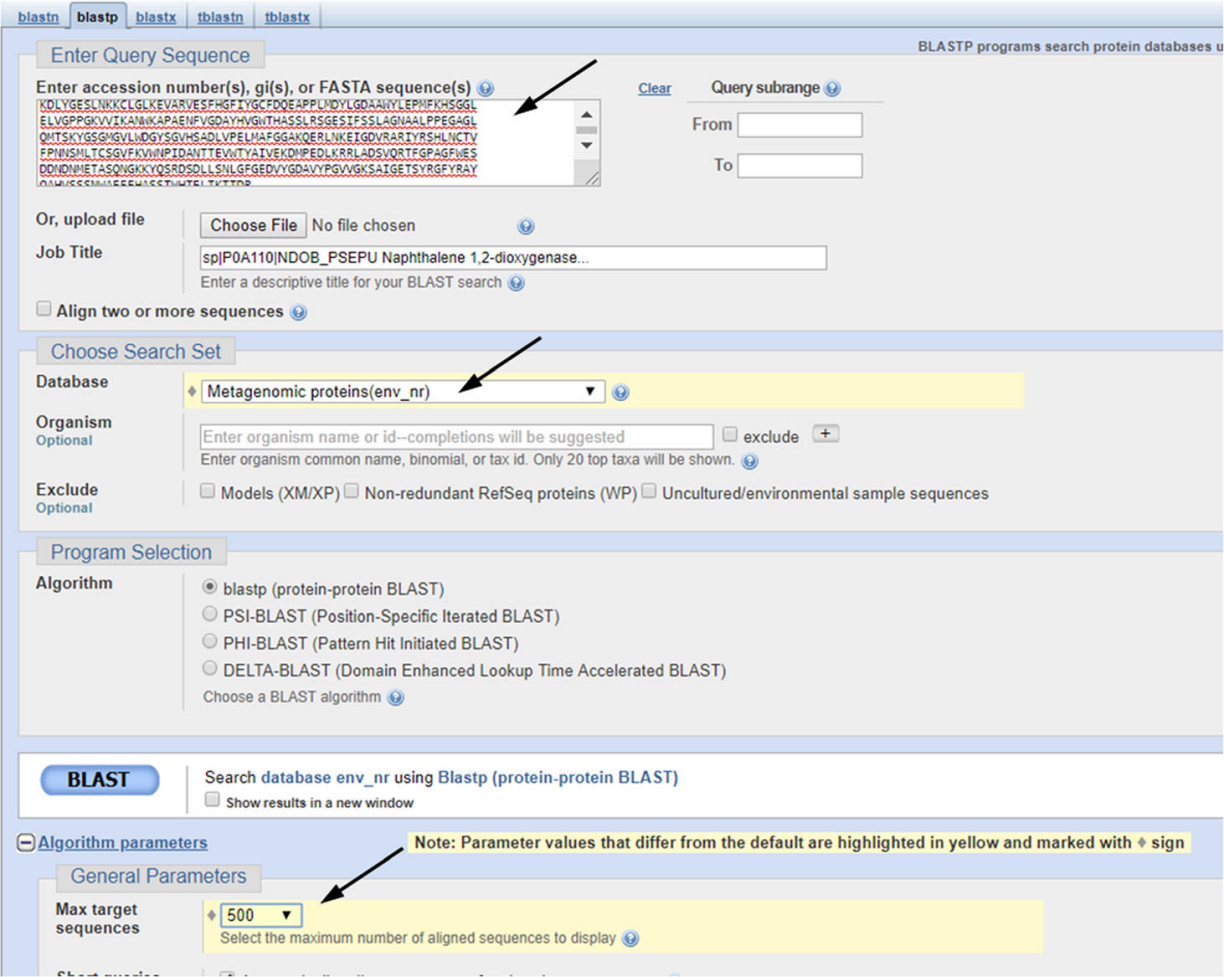
Protein BLAST sheet

Paste the sequence downloaded from UniProt into the “Enter Query Sequence” field, choosing “Metagenomic proteins” as database. In the section “Algorithm parameters”, select 500 as “Max target sequences” and run BLAST (Fig. 9).

**Fig. 10:**
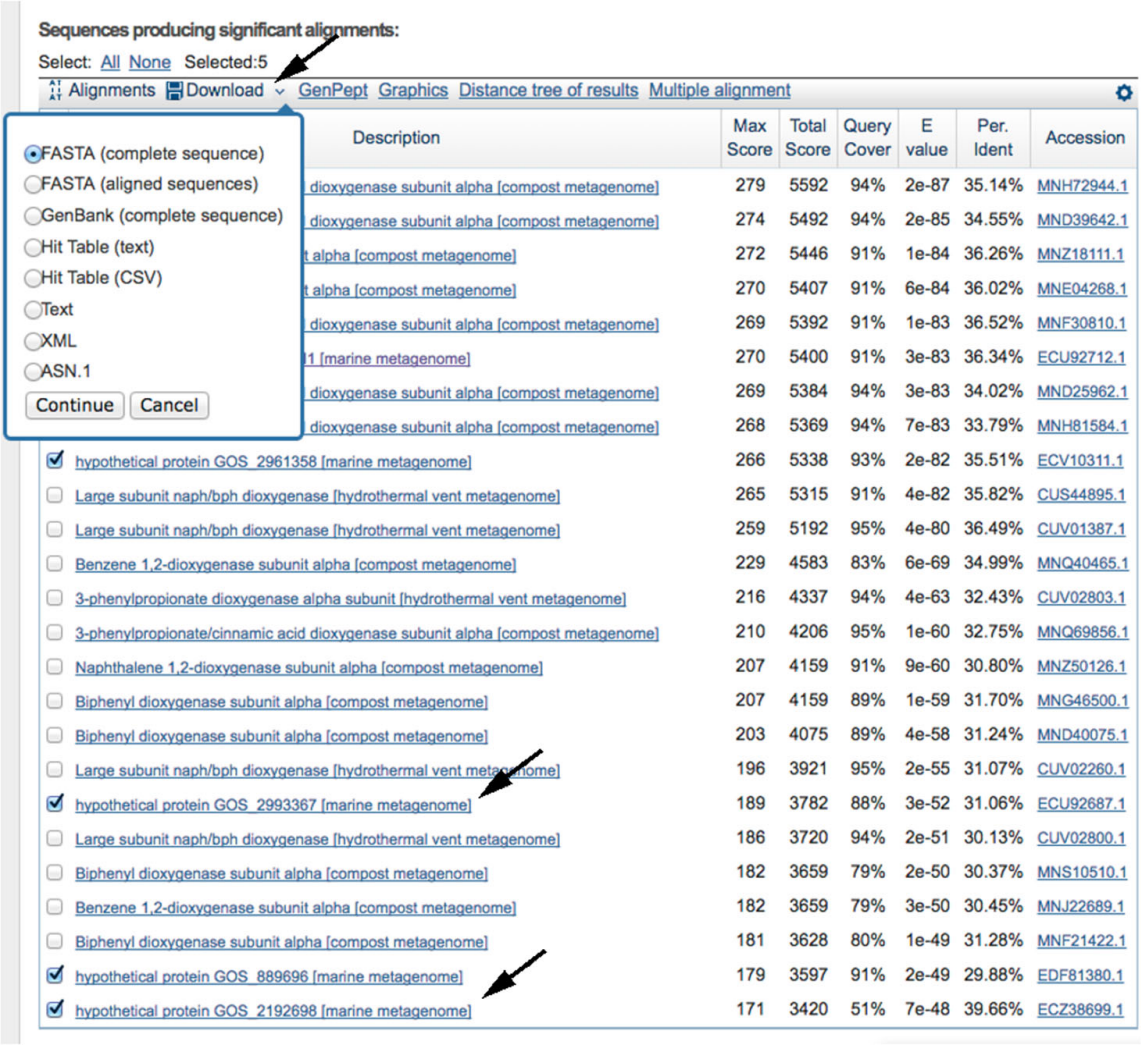
BLASTp output

Select and download all “marine metagenome” sequences in FASTA format (Fig. 10).

#### Step 3. Clustal omega to perform multiple sequence alignment, helpful to predict relations and similarity among sequences

Open a new Internet page with Clustal Omega tool at https://www.ebi.ac.uk/Tools/msa/clustalo/ to perform a multiple alignment.

**Fig. 11:**
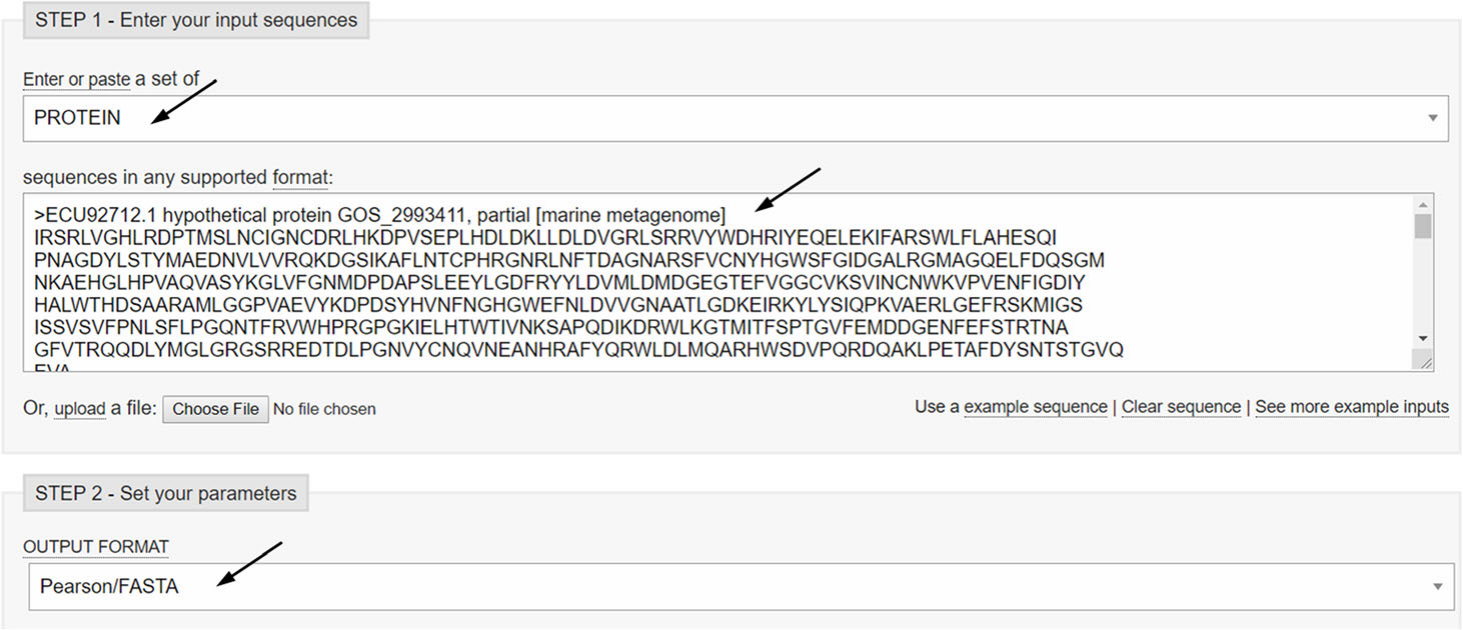
CLUSTAL Omega sheet. Settings for multiple alignment are shown

Paste your sequences in the blank using Pearson/FASTA as output format, leave the other parameters as default and submit the work (Fig. 11).

#### Step 4. Cons: an EMBOSS explorer tool to create a consensus sequence from a multiple alignment

**Fig. 12:**
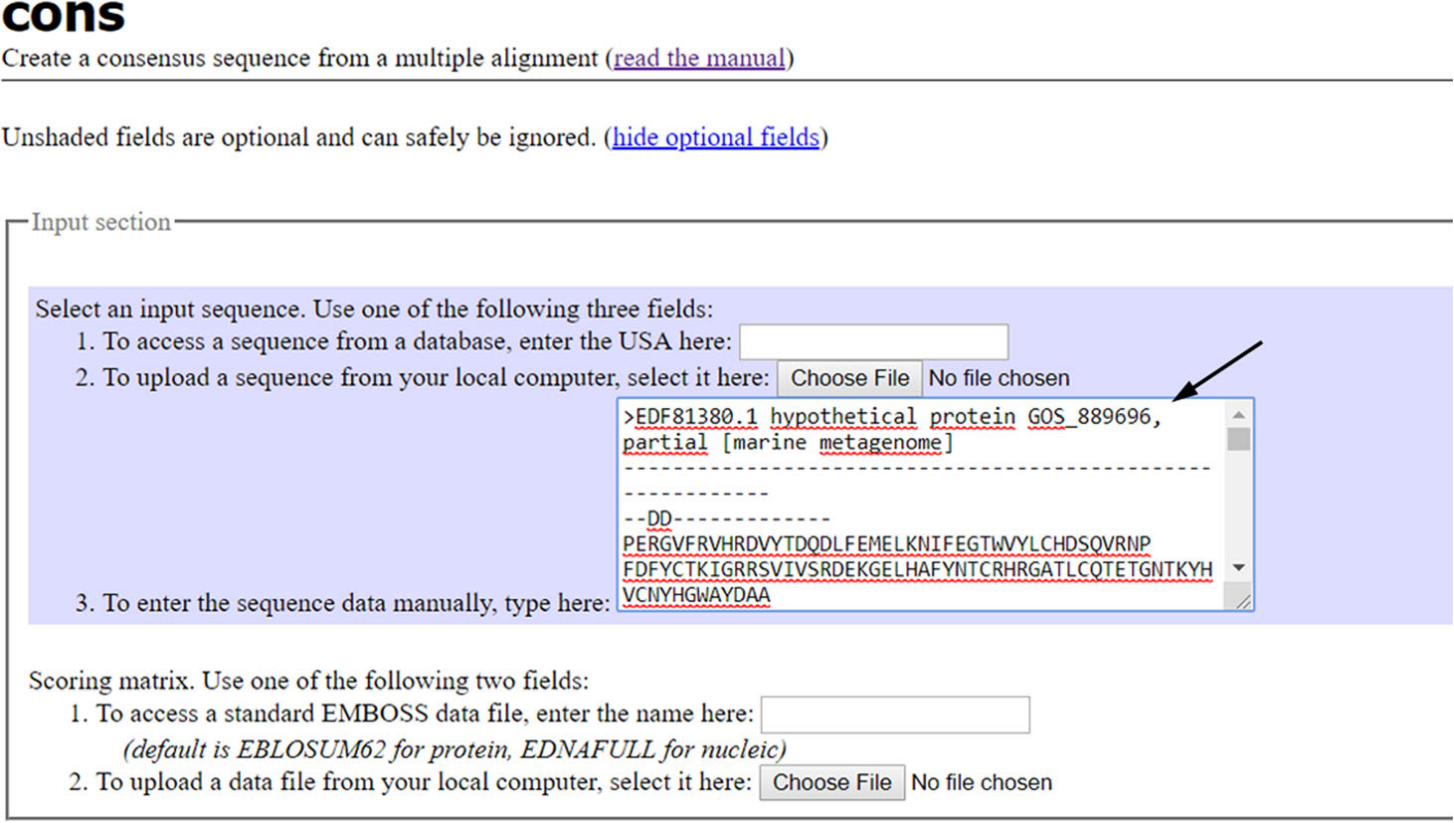
cons interface

**Fig. 13:**
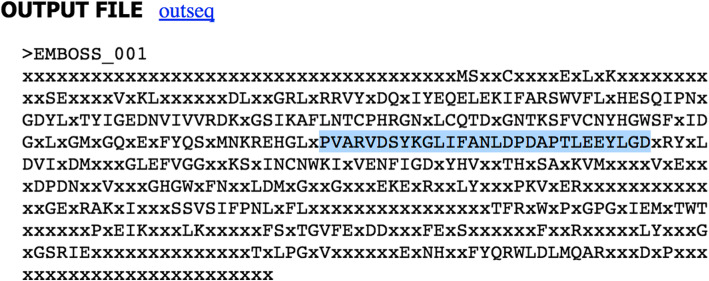
The output file of cons. The figure shows a consensus sequence for Naphthalene 1,2-dioxygenase from marine prokaryotes

Connect to EMBOSS Explorer [35–37] at http://www.bioinformatics.nl/emboss-explorer/ and on the left side of the web sheet click on “cons”. Copy the obtained multiple alignment in FASTA format and paste it in the blank (Fig. 12). An example of consensus sequence output is shown in Fig. 13.

Copy a region (selecting about 30 amino acid residues to obtain a ~ 100 bp DNA probe avoiding regions containing too many “Xs”).

#### Step 5: translate an amino acid sequence into the most probable nucleotide sequence

Search for the web tool Reverse Translate (http://www.bioinformatics.org/sms2/rev_trans.html) to retrotranslate an amino acid sequence into the most probable nucleotide sequence using the default codon usage from *E.coli.*

**Fig. 14:**
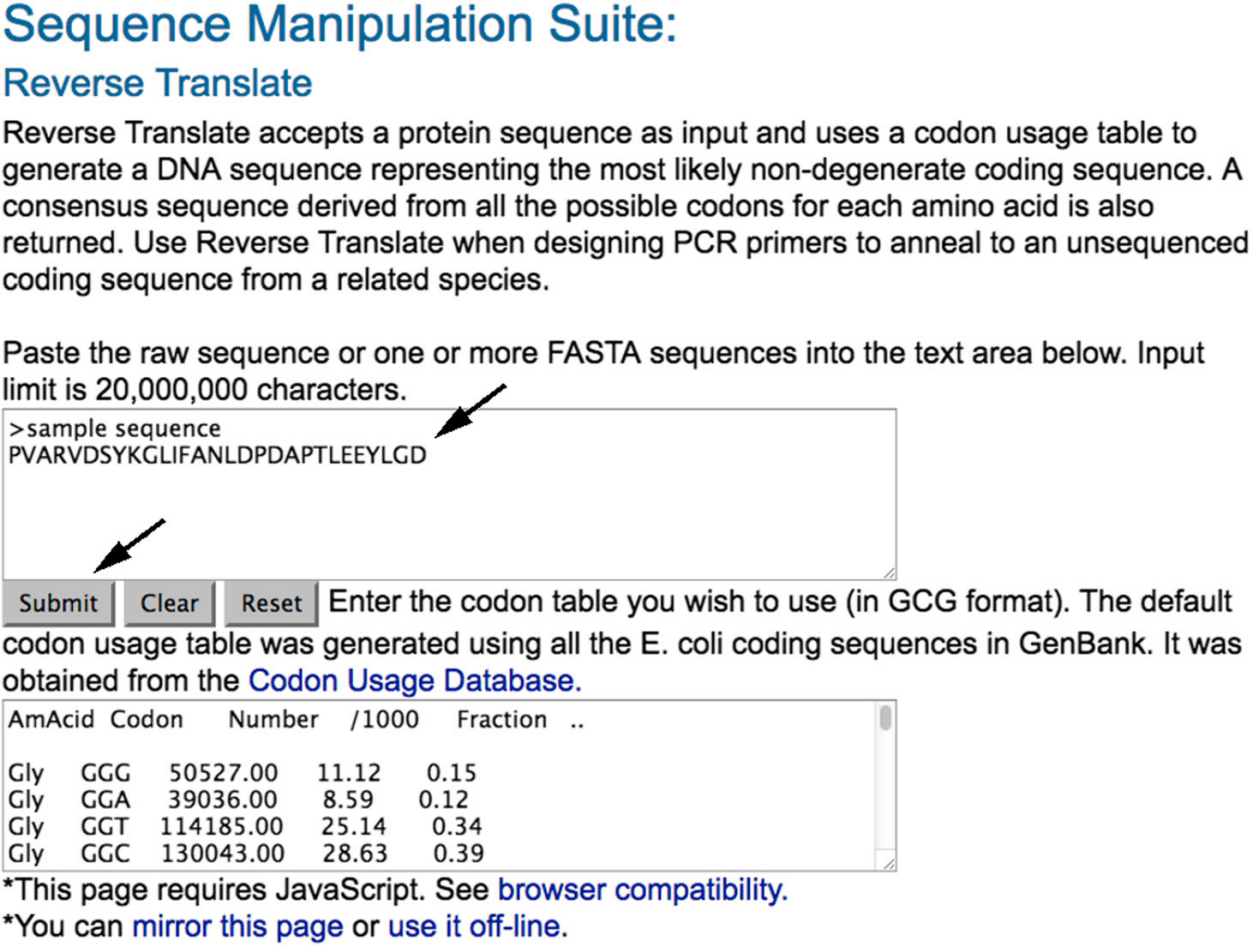
Reverse Translate tool web page

**Fig. 15:**
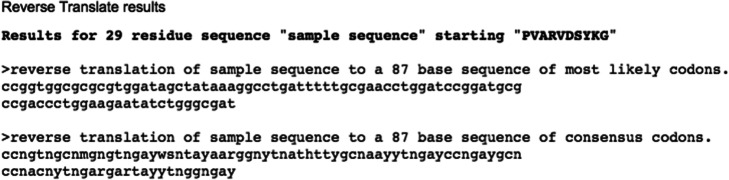
Reverse Translate results. The figure shows the sequence to be used for the screening of the cosmid library

Paste the copied sequence in the blank and submit the work (Fig. 14). In Fig. 15, an example of the obtained nucleotide probe is shown.

## Results

**Fig. 16:**
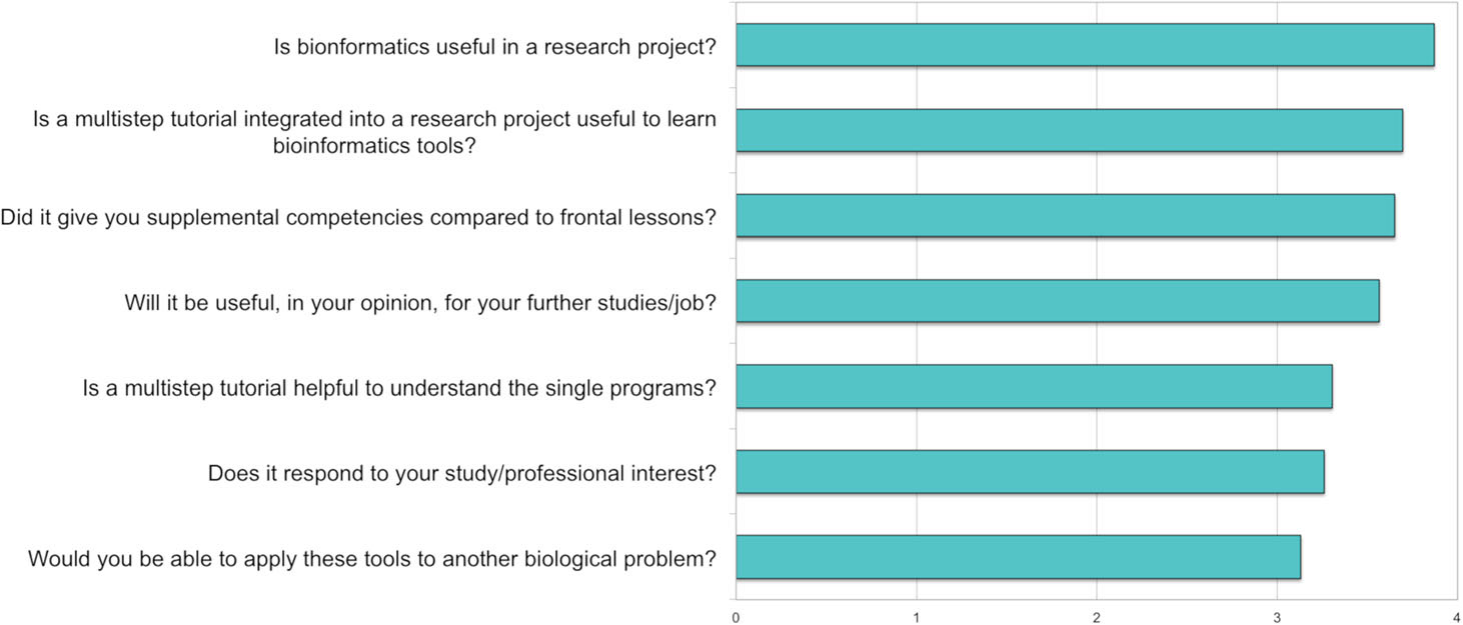
Satisfaction test. The students were posed 7 questions concerning the tutorial and answered with a 0 for “not at all”, 1 for “slightly”, 2 for “moderately”, 3 for “very” and with a 4 for “extremely”. The weighted scores are reported for each score

The tutorial was administered to 23 graduate students who had a bachelor’s degree in biology or natural science with a basic knowledge of biochemistry and molecular biology and no previous knowledge of bioinformatics. This tutorial can be administered to undergraduate students too if they have solid bases of biochemistry and molecular biology. Before administering the tutorial, two lectures were given to introduce the programs and the databases reported in Table 1. All the students without the help of the supervisor concluded the tutorial successfully within one hour. After the practice, students have undergone a satisfaction questionnaire of 7 questions (Additional file 1) to evaluate the general interest and the usefulness of the multistep bioinformatics protocol. All students were satisfied although a minority admitted that they would not be able to apply the same tools to another biological project (30%). Questions indicating the student satisfaction degree are gathered in Fig. 16 and in the supplementary material. Through a computer-based approach, students have been able to search into protein and enzyme databases, performing local and multiple sequence alignments obtaining a consensus sequence and retrofitranslate an amino acid sequence to obtain a DNA sequence.

We are aware of the fact that other types of questionnaires could be administered, for instance, students could be asked to explain the steps that they have performed and why*.*

An even more convincing proof of the usefulness of the tutorial would have been letting the students prepare the cosmid library and, after that, dividing them in two groups. One group would have been asked to screen the library with an enzymatic assay on plates, the second group would have followed the tutorial, designed the probe for colony hybridization and confirmed the activity in the recombinant *E.coli* extracts.

## Discussion

Table 1 summarizes the learning goals of the tutorial. Beyond the immediate ones, i.e. becoming acquainted with very popular bioinformatics tools (Table I) and, in particular, of BRENDA [24], there are far-reaching educational aims. In our opinion the students should recognize the major opportunity offered by big data produced by metagenomics projects and the possibility of deriving protein properties by homology.

The tutorial emphasizes the opportunity offered by metagenomic next-generation sequencing projects. They are like mines we can dig to find what is useful for our research. It is highly probable that proteins homologous to the ones we are interested in are present in the uncharacterized big data stored in databanks.

The tutorial highlights the concept that homologous enzymes share similar functions and that homology can be found comparing sequences. It capitalizes on the principle *‘Pairwise alignments whisper while multiple alignments shout out loud’* (Arthur Lesk). In fact, the students will identify the conserved regions that are a better fit to design a probe carrying out a multiple alignment of proteins homologous to Naphthalene 1,2-dioxygenase from *Pseudomonas putida*.

Consulting BRENDA [24] permits to retrieve original research papers concerning specific classes of enzymes. This will be precious to set up enzymatic assays and check that the cosmid clone isolated by colony hybridization indeed expresses Naphthalene 1,2-dioxygenase.

## Conclusions

Computer-based learning is an excellent method to introduce undergraduate and graduate students with biological and biotechnological background to bioinformatics. We have shown that using bioinformatics tools as steps of a research project is more useful than presenting the same tools separately in stand-alone tutorials and that having a clear experimental objective, possibly related to a “trendy” topic such as green economy [38] raises the students’ interest.

## Supplementary information

**Additional file 1.** Student Satisfaction Questionnaire.docx

## Data Availability

The programs used during the current practice are available at: BENDA, https://www.brenda-enzymes.org/index.php Accessed 03/09/2019 BLASTP, https://blast.ncbi.nlm.nih.gov/Blast.cgi Accessed 03/09/2019 UniProt, http://www.uniprot.org/ Accessed 03/09/2019 Clustal Omega, https://www.ebi.ac.uk/Tools/msa/clustalo/ Accessed 03/09/2019 Cons, http://www.bioinformatics.nl/emboss-explorer/ Accessed 03/09/2019 Reverse translate, http://www.bioinformatics.org/sms2/rev_trans.html Accessed 03/09/2019

## References

[1] Campbell AM (2003). Public access for teaching genomics, proteomics, and bioinformatics. Cell Biol Educ.

[2] Pevzner P, Shamir R (2009). Computing has changed biology—biology education must catch up. Science.

[3] Letchford J, Corradi H, Day T (2017). A flexible e-learning resource promoting the critical reading of scientific papers for science undergraduates. Biochem Mol Biol Educ.

[4] Cimmaruta C, Liguori L, Monticelli M, Andreotti G. Citro V. E-Learning for Rare Diseases: An Example Using Fabry Disease. Int J Mol Sci. 2017;18(10):2049. 10.3390/ijms18102049 PMC5666731 28946642

[5] King MD, Phillips P, Turner MW, Katz M, Lew S, Bradburn S, Andersen T, McDougal OM (2016). Computational exploration of a protein receptor binding space with student proposed peptide ligands. Biochem Mol Biol Educ.

[6] Korcsmaros T, Dunai ZA, Vellai T, Csermely P (2013). Teaching the bioinformatics of signaling networks: an integrated approach to facilitate multi-disciplinary learning. Brief Bioinform.

[7] Ray S, Koshy NR, Reddy PJ, Srivastava S (2012). Virtual labs in proteomics: new E-learning tools. J Proteome.

[8] Kossida S, Tahri N, Daizadeh I (2002). Bioinformatics by example: from sequence to target. J Chem Educ.

[9] Blatter M-C, Baillie Gerritsen V, Palagi PM, Bougueleret L. Xenarios I. The Metagenomic Pizza: a simple recipe to introduce bioinformatics to the layman. EMBnet.journal. 2016;22:e864.

[10] Hingamp P, Brochier C, Talla E, Gautheret D, Thieffry D, Herrmann C (2008). Metagenome annotation using a distributed grid of undergraduate students. PLoS Biol.

[11] Gibbens BB, Scott CL, Hoff CD, Schottel JL (2015). Exploring metagenomics in the laboratory of an introductory biology course. J Microbiol Biol Educ.

[12] Edwards RA, Haggerty JM, Cassman N, Busch JC, Aguinaldo K, Chinta S, Vaughn MH, Morey R, Harkins TT, Teiling C (2013). Microbes, metagenomes and marine mammals: enabling the next generation of scientist to enter the genomic era. BMC Genomics.

[13] Quatrini R, Valdès J, Jedlicki E, Holmes DS. The use of bioinformatics and genome biology to advance our understanding of bioleaching microorganisms. In: Donati E.R., Sand W. (eds). Microbial Processing of Metal Sulfides. Springer, Dordrecht. 2007:221–39.

[14] Welsch ME, Snyder SA, Stockwell BR (2010). Privileged scaffolds for library design and drug discovery. Curr Opin Chem Biol.

[15] Qu Y, Shen E, Ma Q, Zhang Z, Liu Z, Shen W, Wang J, Li D, Li H, Zhou J (2015). Biodegradation of indole by a newly isolated Cupriavidus sp. SHE. J Environ Sci (China).

[16] Gibson DT, Parales RE (2000). Aromatic hydrocarbon dioxygenases in environmental biotechnology. Curr Opin Biotechnol.

[17] Kennedy J, Flemer B, Jackson SA, Lejon DP, Morrissey JP, O'Gara F, Dobson AD (2010). Marine metagenomics: new tools for the study and exploitation of marine microbial metabolism. Mar Drugs.

[18] Rath CM, Janto B, Earl J, Ahmed A, Hu FZ, Hiller L, Dahlgren M, Kreft R, Yu F, Wolff JJ (2011). Meta-omic characterization of the marine invertebrate microbial consortium that produces the chemotherapeutic natural product ET-743. ACS Chem Biol.

[19] Subramani R, Aalbersberg W (2012). Marine actinomycetes: an ongoing source of novel bioactive metabolites. Microbiol Res.

[20] Madhavan A, Sindhu R, Parameswaran B, Sukumaran RK, Pandey A (2017). Metagenome analysis: a powerful tool for enzyme bioprospecting. Appl Biochem Biotechnol.

[21] Popovic A, Tchigvintsev A, Tran H, Chernikova TN, Golyshina OV, Yakimov MM, Golyshin PN, Yakunin AF: Metagenomics as a Tool for Enzyme Discovery: Hydrolytic Enzymes from Marine-Related Metagenomes. In: Prokaryotic Systems Biology*.* Edited by Krogan PNJ, Babu PM. Cham: Springer International Publishing; 2015: 1–Metagenomics as a Tool for Enzyme Discovery: Hydrolytic Enzymes from Marine-Related Metagenom20. 10.1007/978-3-319-23603-2_1 26621459

[22] Alma’abadi AD, Gojobori T, Mineta K (2015). Marine Metagenome as a resource for novel enzymes. Genomics, Proteomics Bioinformatics.

[23] Ufarté L, Laville É, Duquesne S, Potocki-Veronese G (2015). Metagenomics for the discovery of pollutant degrading enzymes. Biotechnol Adv.

[24] BRENDA: [https://www.brenda-enzymesorg] 2019.Accessed 03 Sept 2019.

[25] Schomburg I, Jeske L, Ulbrich M, Placzek S, Chang A, Schomburg D (2017). The BRENDA enzyme information system-from a database to an expert system. J Biotechnol.

[26] Johnson M, Zaretskaya I, Raytselis Y, Merezhuk Y, McGinnis S, Madden TL (2008). NCBI BLAST: a better web interface. Nucleic Acids Res.

[27] Laursen L (2011). Spain's ship comes in. Nature.

[28] Rusch DB, Halpern AL, Sutton G, Heidelberg KB, Williamson S, Yooseph S, Wu D, Eisen JA, Hoffman JM, Remington K (2007). The sorcerer II Global Ocean sampling expedition: Northwest Atlantic through eastern tropical Pacific. PLoS Biol.

[29] Sunagawa S, Karsenti E, Bowler C, Bork P (2015). Computational eco-systems biology in Tara oceans: translating data into knowledge. Mol Syst Biol.

[30] Stothard P (2000). The sequence manipulation suite: JavaScript programs for analyzing and formatting protein and DNA sequences. Biotechniques.

[31] The UniProt Consortium (2019). UniProt: a worldwide hub for protein knowledge. Nucleic Acids Res.

[32] Resnick S, Lee K, Gibson D (1996). Diverse reactions catalyzed by naphthalene dioxygenase fromPseudomonas sp strain NCIB 9816. J Ind Microbiol.

[33] Parales JV, Kumar A, Parales RE, Gibson DT (1996). Cloning and sequencing of the genes encoding 2-nitrotoluene dioxygenase from Pseudomonas sp. JS42. Gene.

[34] Sievers F, Higgins DG (2018). Clustal omega for making accurate alignments of many protein sequences. Protein Sci.

[35] Mullan LJ, Bleasby AJ (2002). Short EMBOSS user guide. European molecular biology open software suite. Brief Bioinform.

[36] Olson SA (2002). EMBOSS opens up sequence analysis. European molecular biology open software suite. Brief Bioinform.

[37] Rice P, Longden I, Bleasby A (2000). EMBOSS: the European molecular biology open software suite. Trends Genet.

[38] Płotka-Wasylka J, Kurowska-Susdorf A, Sajid M, de la Guardia M, Namieśnik J, Tobiszewski M (2018). Green chemistry in higher education: state of the art, challenges, and future trends. ChemSusChem.

