# Supplementary material for: Bioinformatics tools for marine biotechnology: a practical tutorial with a metagenomic approach
Source: BMC Bioinformatics. 2020 Aug 21;21(Suppl 10):348. doi: 10.1186/s12859-020-03560-z (PMC7447578; doi:10.1186/s12859-020-03560-z)
Supplement: Supplementary file 1 — Additional file 1. Student Satisfaction Questionnaire.docx [file 12859_2020_3560_MOESM1_ESM.docx]

**STUDENT SATISFACTION QUESTIONNAIRE**

Please mark the number from the evaluation options that is closest to your personal experience.

0=not at all

1=slightly

2=moderately

3=very

4=extremely

1. Is bioinformatics useful in a research project?

0□ 1□ 2□ 3□ 4□

1. Is a multistep tutorial helpful to understand the single programs?

0□ 1□ 2□ 3□ 4□

1. Is a multistep tutorial integrated into a research project useful to learn bioinformatics tools?

0□ 1□ 2□ 3□ 4□

1. Did it give you supplemental competencies compared to frontal lessons?

0□ 1□ 2□ 3□ 4□

1. Would you be able to apply these tools to another biological problem? 0□ 1□ 2□ 3□ 4□
2. Does it respond to your study/professional interest?

0□ 1□ 2□ 3□ 4□

1. Will it be useful, in your opinion, for your further studies/job?

0□ 1□ 2□ 3□ 4□
